# In Vivo Confocal Microscopy of Trachoma in Relation to Normal Tarsal Conjunctiva

**DOI:** 10.1016/j.ophtha.2010.08.029

**Published:** 2011-04

**Authors:** Victor H. Hu, Patrick Massae, Helen A. Weiss, Ian A. Cree, Paul Courtright, David C.W. Mabey, Robin L. Bailey, Matthew J. Burton

**Affiliations:** 1 International Centre for Eye Health, Department of Infectious and Tropical Diseases, London School of Hygiene and Tropical Medicine, London, United Kingdom; 2 Kilimanjaro Centre for Community Ophthalmology, Moshi, Tanzania; 3 MRC Tropical Epidemiology Group, London School of Hygiene and Tropical Medicine, London, United Kingdom; 4 Department of Pathology, Institute of Ophthalmology, University College London, United Kingdom; 5 Clinical Research Unit, Department of Infectious and Tropical Diseases, London School of Hygiene and Tropical Medicine, London, United Kingdom

## Abstract

**Objective:**

To describe the in vivo confocal microscopy (IVCM) appearances of the tarsal conjunctiva in trachoma compared with the appearance of healthy conjunctiva and to develop grading systems for IVCM examination of the tarsal conjunctiva for use in future studies on trachoma and other conjunctival diseases.

**Design:**

Prospective observational study.

**Participants:**

In vivo confocal microscopy examination was performed on 302 clinically normal adults, 16 clinically normal children, 750 adults with trachomatous conjunctival scarring, and 25 children with active trachoma.

**Methods:**

Clinical evaluation was performed with ×2.5 loupes, and IVCM examination of the upper tarsal conjunctiva was carried out with a Heidelberg Retina Tomograph 3 with the Rostock Cornea Module (Heidelberg Engineering GmbH, Dossenheim, Germany).

**Main Outcome Measures:**

In vivo confocal microscopy images were analyzed for cellular and tissue changes associated with trachomatous inflammation and scarring compared with healthy subjects.

**Results:**

Trachomatous subjects with follicular and papillary inflammation had an increased inflammatory cellular infiltrate, including dendritiform cells, discrete follicular and papillary structures, and cystic lacunae suggestive of tissue edema. Trachomatous conjunctival scarring was seen with IVCM as organization of the subepithelial connective tissue into bands/sheets. Grading systems for inflammatory changes and scarring were developed, with the system for scarring showing good interobserver agreement with an intraclass coefficient of 0.88.

**Conclusions:**

In vivo confocal microscopy provides a powerful tool for examining the ocular surface. Numerous cellular and tissue changes were observed in subjects with trachoma, the first time IVCM has been applied to this disease. These changes both complement and add to previous histologic analyses. In vivo confocal microscopy promises to provide new insights into the pathogenesis of trachoma and other conjunctival diseases.

**Financial Disclosure(s):**

The author(s) have no proprietary or commercial interest in any materials discussed in this article.

Trachoma is caused by recurrent infection with *Chlamydia trachomatis*. The infection is most commonly found in children who develop a chronic follicular conjunctivitis with papillary hypertrophy, referred to as “active trachoma.” Later in life, such individuals are at risk of developing conjunctival scarring, entropion, trichiasis, and eventually blinding corneal opacification. Trachoma is an ancient disease that was previously endemic in Europe and North America but is now largely found in poor rural communities in the developing world. Trachoma remains the most common infectious cause of blindness globally with more than 1.3 million people blind from the disease, 8.2 million with trichiasis, and 40 million with active disease.^1,2^

In vivo confocal microscopy (IVCM) is a noninvasive means of obtaining high-resolution tissue images down to the cellular level. The illumination and observation systems are focused on the same focal point, giving rise to the term “confocal,” so that any light originating from outside the focal plane is highly suppressed. This allows a magnification of up to 800× with an optical resolution of 1 to 2 μm. This relatively new technique has shown promise in the study of ocular surface disease, including corneal dystrophies, changes after refractive surgery, drug toxicity, and dendritic cell changes in inflammatory disease.^3–5^ The technique's application to the study of ocular infections has shown that it is able to differentiate among bacterial, fungal, and protozoan agents.^6^ Although much of the work done so far has been concentrated on the cornea, several studies have reported interesting results on the conjunctiva, including changes in atopic keratoconjunctivitis, filtration blebs after glaucoma surgery, and inflammatory changes in response to bacterial lipopolysaccharide.^3,7–11^

In vivo confocal microscopy has several advantages over alternative methods of studying cellular events in the conjunctiva. Histopathology of biopsy specimens, the “gold standard,” allows exceptional detail to be seen, but it is an invasive procedure in which usually only a small amount of tissue can be investigated and sequential examination over time is not feasible. In addition, there can be artefact changes in the tissue introduced during processing. Impression cytology can be useful; however, cells are probably collected from only the most superficial layers of the epithelium, and no information about the structure of the tissue is provided. In vivo confocal microscopy has not been associated with any adverse events, can be used repeatedly all over the ocular surface, and provides high-resolution images.

The pathogenesis of trachoma is poorly understood. The infection itself is believed to be limited to a small minority of the superficial conjunctival epithelial cells. It is generally accepted that much of the tissue damage in this disease is the result of a pathologic immune response.^12–17^ A limited serovar-specific protective immunity probably develops. However, it remains unclear why some individuals develop blinding sequelae and others do not (with a similar infection exposure), and what factors are important in the progression of scarring. In vivo confocal microscopy has the potential to provide useful insights into this disease process at the tissue level. Specifically, IVCM can reveal the morphology and distribution of fibrotic tissue within the palpebral conjunctiva. In addition, inflammatory cell infiltrates can be examined and related to the clinical phenotype.

This article presents descriptive findings from IVCM of the different clinical stages of trachoma and compares these with normal tarsal conjunctiva. We also propose a grading system for IVCM findings in subjects with trachoma to facilitate formal image analysis in ongoing studies. It is anticipated that this grading system will also have relevance for other immuno-fibrogenic diseases of the conjunctiva, such as mucous membrane pemphigoid and atopic keratoconjunctivitis.

## Patients and Methods

### Ethical Permission

The observations presented were made during the course of studies approved jointly by the London School of Hygiene and Tropical Medicine Ethics Committee, the Kilimanjaro Christian Medical Centre Ethics Committee, and the National Ethics Committee of the Tanzanian National Institute of Medical Research. Informed consent was obtained before enrollment of each subject.

### Clinical Assessment

These studies were conducted in trachoma endemic communities in Siha District, Kilimanjaro Region, Northern Tanzania. Subjects were examined in a dark room or tent with ×2.5 loupes and a bright torch. Signs of trachoma were graded using the 1981 detailed World Health Organization grading system, which assesses the upper palpebral conjunctiva for follicles, papillae, and scarring, and grades entropion/trichiasis and corneal opacity.^18^ A portable slit lamp was used if a more magnified view was needed, particularly to rule out subtle conjunctival scarring in normal controls. High-resolution digital photographs were taken of the upper tarsal conjunctiva.

The protocols for the ongoing related studies involved the recruitment of 800 adults with trachomatous conjunctival scarring and 360 adults without scarring. We attempted IVCM examinations on all consenting individuals. In a previously untreated village that was about to receive mass drug administration with azithromycin for trachoma control, we also performed IVCM examinations on children who could comfortably tolerate the procedure.

### Confocal Microscopy Assessment

In vivo confocal microscopy was performed using the Heidelberg Retina Tomograph 3 (HRT3) in combination with the Rostock Cornea Module (RCM) (Heidelberg Engineering GmbH, Dossenheim, Germany). This uses a 670-nm diode laser as a light source with a ×63 water-contact objective (Olympus Europa GmbH, Hamburg, Germany) covered with a sterile single-use polymethylmethacrylate (PMMA) cap (Tomocap, Heidelberg Engineering). A small amount of carbomer gel (GelTears, Chauvin Pharmaeuticals Ltd., Surrey, UK) was used as a coupling agent between the RCM lens and the cap. The device scans an area of 400×400 μm with a magnification of ×800 and a lateral resolution of 1 μm.

The related study protocols required confocal examination of the upper tarsal conjunctiva in the left eye in adults and the right eye in children. Topical anesthesia was applied to the conjunctival sac (proxymetacaine 0.5%, Chauvin Pharmaceuticals Ltd., Surrey, UK), and the upper eyelid was everted. Additional coupling gel was not needed for the examination of the conjunctiva. The position of the HRT3/RCM unit was adjusted to bring the surface of the device into contact with the tarsal portion of the palpebral conjunctiva, with the assistance of a live-view side video camera. Scans were taken using the “volume” setting in which 40 coronal images are taken in rapid succession at 2.1-μm intervals from superficial to deep. Scans started at the conjunctival epithelial surface, and the final scan was at a depth of 85 μm. It was generally found that the quality of the image became impaired beyond 85 μm with little structural detail visible. The brightness control was set on automatic. Ten volume scans were taken per patient from random locations across the tarsal conjunctiva. The total examination time was approximately 5 minutes. In selected patients, scans of the marginal portion of the palpebral conjunctiva were also obtained in a manner similar to those of the tarsal conjunctiva. None of the IVCM images shown in this article have had any adjustments made (e.g., in contrast or brightness) after being exported from the HRT3/RCM unit.

## Results

### Study Subjects

All IVCM examinations were performed as part of studies performed in a trachoma endemic area in Siha District of northern Tanzania. The descriptions of normal and diseased conjunctiva and the proposed grading systems are based on experience gained from IVCM examinations of 302 clinically normal adults (≥18 years), 16 clinically normal children (mean age 9.4 years, range 5–15 years), 750 adults with a wide range of trachomatous conjunctival scarring, and 25 children with active trachoma (mean 6.8 years, range 3–17 years).

### Normal Palpebral Conjunctiva

**Figure 1:**
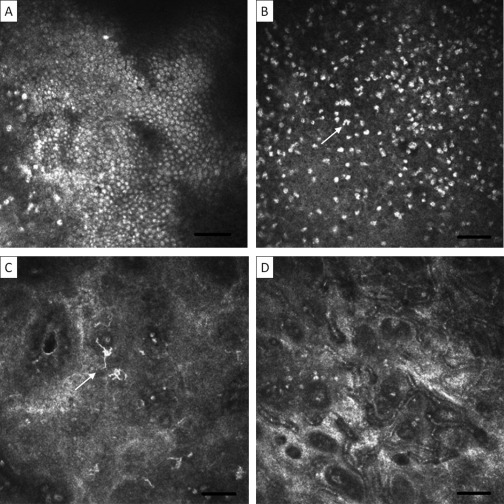
Normal tarsal conjunctiva. Images are 400×400 μm with the bar representing 50 μm. **A**, Superficial epithelial cell nuclei. **B**, Inflammatory cell nuclei; note heterogeneity in size and shape (*arrow*). **C**, Dendritic cells (*arrow*). **D**, Superficial blood vessels.

In the tarsal portion of the healthy palpebral conjunctiva, round grey bodies can be seen at the surface, which probably represent epithelial cell nuclei (Fig 1A; further images of normal conjunctiva can also be seen in Fig 2, available at http://aaojournal.org). These are not always readily seen, and it is difficult to determine how many layers there are with IVCM. Scattered among the epithelial cells and extending beneath them are bright bodies (Fig 1B), which have been described as inflammatory cell nuclei. These are heterogeneous in size and shape, with some being circular and others multi-lobulated. These scattered presumed inflammatory cells are present to a depth of approximately 20 μm. Dendritiform cells (DCs) are sometimes seen near the surface of the conjunctiva (Fig 1C). These appear as bright structures with cell bodies and multiple processes. In clinically normal conjunctiva, these processes are relatively short and there is usually no interdigitation with those of other DCs.

Round black spaces are often seen in the superficial conjunctiva, previously described as “microcysts” (Fig 2F, available at http://aaojournal.org). Sequential scanning shows these to be tubular in shape. The spaces contain highly reflective material, often in the form of discrete, round particles or cells. There is also often a bright reflection around their edge, suggestive of encapsulation. Approximately 20 to 30 μm below the surface, a fine network of small blood vessels is usually seen (Fig 1D). These are surrounded by brightly reflective fibrous material that is probably supportive connective tissue. Beneath the small blood vessels, there is loose connective tissue within which few, if any, cells are seen (see Conjunctival Scarring section below). Deep blood vessels, which are much broader than the more superficial network, can be seen at variable depths with blood flowing inside (Fig 2H, available at http://aaojournal.org). Sometimes an oblique view is obtained (as opposed to the usual *en face*), as a result of unequal apposition of the PMMA cap on the conjunctival surface, which gives some appreciation of the various strata in the tissue (Fig 2I, available at http://aaojournal.org).

If confocal microscopy is performed on the marginal portion of the palpebral conjunctiva, next to the lid margin, then adenoid structures can be seen, which probably represent meibomian gland ducts or acinar units (Fig 2J, available at http://aaojournal.org). The IVCM appearance of palpebral conjunctiva in children and young adults is similar to that of older adults. One feature observed in the conjunctiva of approximately 20% of children (both clinically normal and inflamed) was fine blood vessels seen in cross-section (Fig 2K, available at http://aaojournal.org). Unlike the other small vessels mentioned above, which are parallel to the conjunctival surface, these vessels are perpendicular. They extended from a very superficial level to around the level of the usual vascular network at 20 μm and were surrounded with highly reflective connective tissue.

### Active Trachoma in Children

All of the children with active trachoma examined by IVCM were grade F2 or P2 on the World Health Organization grading system (i.e., having a significant number of follicles or papillary inflammation). Follicular structures occasionally were found (Fig 3A, available at http://aaojournal.org), which were formed of discrete collections of moderate to highly reflective round cell nuclei. They began at approximately 10 μm below the surface and were roughly spherical with an estimated maximum diameter of approximately 0.5 to 1 mm.

**Figure 4:**
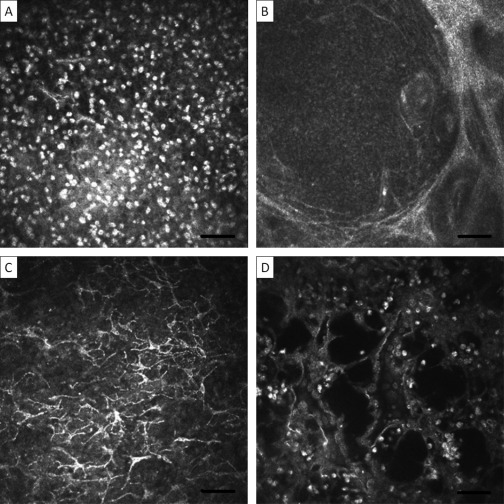
Active disease in adults with conjunctival scarring. Images are 400×400μm with the bar representing 50 μm. **A**, Increased inflammatory cell infiltrate. **B**, Follicular structure. **C**, Activated DCs. **D**, Cystic lacunae. DC = dendritiform cells.

Approximately half of the children with active disease had black, “cystic” spaces or lacunae near the conjunctival surface, which may reflect areas of tissue edema (Fig 3B, available at http://aaojournal.org; corresponding image from an adult is shown in Fig 4D). These lacunae were generally larger and more irregular in outline than the microcystic spaces seen in normal conjunctiva, and they did not have the surrounding bright edge, suggestive of encapsulation. These spaces were largely acellular with no flow seen, which suggests that they are not blood vessels. Approximately half of the children also had a network or honeycomb of interconnecting bands (Fig 3C, available at http://aaojournal.org). This network was seen near the surface and extended into the tissue for a variable distance. Small vessels were often seen near the surface in the central areas of this network, and it is likely that this appearance represents tiny papillae. Dendritiform cells were seen more frequently in children with active disease and, when present, were more numerous and had longer dendritic processes that often interdigitated (Fig 3D, available at http://aaojournal.org).

### Trachomatous Inflammation in Adults with Conjunctival Scarring

Clinical inflammation is often seen in adults with trachomatous scarring. In adults with clinical inflammation and scarring, IVCM often reveals an increase in the inflammatory cell infiltrate that was more marked than in children with active disease (Fig 4A; further images of conjunctiva from adults with inflammation and scarring can also be seen in Fig 5, available at http://aaojournal.org). This increased cellularity is most prominent in the superficial conjunctiva, rarely extending beyond 20 μm. Papilliform structures, composed of discrete conjunctival elevations with central vessels were seen with IVCM (Fig 5B, available at http://aaojournal.org). Follicular structures (<0.5 mm) were also observed in some subjects (Fig 4B), although follicles were rarely seen clinically. Cystic lacunae and DCs were also seen in adults (Fig 4C, D). The honeycomb of interconnecting bands was rarely seen.

For the evaluation of markers of inflammatory activity, we propose the grading scheme shown in Table 1. There was some variation in the inflammatory cell count density between images taken from different areas of the same conjunctival surface. For 30 subjects, we measured the cell count in the full 10 volume scans taken for each subject. From the variation in these estimates from the same lid, we found that the average cell count score of 3 random images provided sufficient precision.

### Conjunctival Scarring

**Figure 6:**
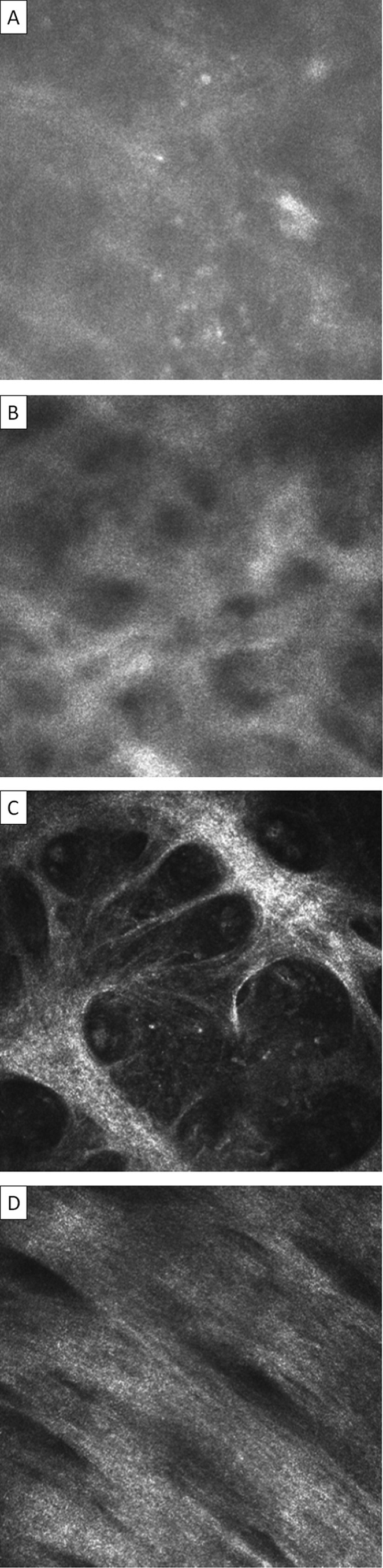
Conjunctival connective tissue organization/scarring grading system for IVCM. Images are 400×400 μm. **A**, Normal: homogenous, amorphous appearance with occasional fine, wispy strand. **B**, Grade 1: Heterogeneous appearance with poorly defined clumps or bands present. **C**, Grade 2: clearly defined bands of tissue that constitute <50% of the area of the scan. **D**, Grade 3: clearly defined bands or sheets of tissue that constitute ≥50% of the area of the scan and in which striations are visible. If different grades of scarring are seen within a particular volume scan, then the highest grade is recorded. The connective tissue that is graded needs to be separate from that associated with the vascular tissue; if this is not possible then the scan is considered ungradable. IVCM = in vivo confocal microscopy.

The appearance of the subepithelial connective tissue (starting at ∼30 μm from the surface) in individuals with clinically visible trachomatous conjunctival scarring varied widely. At the mild end of the spectrum, the appearance was similar to that seen in normal controls: mostly moderately reflective, amorphous tissue with a few fine, wispy strands randomly arranged. With increasing severity of clinically visible scarring, increasingly marked changes were found in the deeper subepithelial connective tissue. In the more severe cases, we observed broad bands of highly reflective connective tissue that were often arranged in parallel, with a striated “texture.” We interpret this appearance as organized bands of scar tissue. By reviewing these images, we developed a 4-point grading system for the degree of subepithelial connective tissue organization/scarring: normal to grade 3. Definitions and characteristic examples are provided in Figures 6 and 7 (available at http://aaojournal.org).

An overall IVCM connective tissue organization/scarring grade can be calculated with this grading system. Each volume scan is given a score of 0 (normal), 1 (grade 1), 2 (grade 2), or 3 (grade 3). If the grade varies between the individual images of the volume scan, then the highest grade is used. The mean score for that patient is then calculated by dividing the sum of the volume scan scores by the number of volume scans read. At least 3 gradable volume scans need to be available to generate a mean score. The minimum score possible is therefore 0 and the maximum is 3 (if each volume scan score was 0 or 3, respectively).

Interobserver agreement was assessed by calculating the intraclass correlation coefficient on the mean scores of 50 patients. These were graded independently by VH and MB, who were also masked to the clinical appearance. The intraclass correlation coefficient showed a high agreement of 0.88, that is, 88% of the total variance was due to between individual variation (rather than between observer variation).

As described in the methodology, up to 10 sets of volume scans were taken from each subject. We found that the scarring grade generally showed little variation between the multiple images taken from different areas of the same tarsal conjunctiva. Analysis of the image grading of 50 randomly selected subjects with conjunctival scarring showed that all the separate volume scans of 74% of individuals had a difference of ≤1 (i.e., all scores were within 1 grade of each other). Only 1 individual had a range of 3.

Meibomian glands were not systematically imaged as part of the study protocols. However, acinar units at the marginal epithelium were seen in a few subjects with trachomatous scarring. In a limited number of scans, the lumen of these acinar units was seen to contain low reflectivity material, possibly scar tissue (Fig 8A, available at http://aaojournal.org). The walls of the units were also poorly defined, in contrast with those from normal subjects. In subjects with more advanced scarring, concretions were also a frequent finding (Fig 8B, available at http://aaojournal.org).

## Discussion

The pathogenesis of trachoma has been studied from various angles, but these have tended not to show what is happening at a tissue or cellular level.^19–22^ A number of studies have used histopathology and immunohistochemistry. However, these involve an invasive biopsy procedure and analysis is restricted to a tiny amount of tissue.^23^ We have carried out a large number of IVCM examinations in both trachomatous and clinically non-scarred subjects to gain insights into the pathophysiology of progressive conjunctival scarring in trachoma. To the best of our knowledge, this is the first time IVCM has been reported from individuals with trachoma or from children.

Follicular and papillary inflammation are the key features of active trachoma. Follicular structures can be seen with IVCM in the subepithelial tissue, probably composed of the nuclei of inflammatory cells and corresponding to follicles seen clinically. Histologic analysis has shown these to be composed mainly of B cells with some macrophages and T cells.^23,24^ Follicles in adults may be seen on histology without being a prominent clinical feature, and these follicles seem to be morphologically distinct from those in children in that they lack germinal centers.^25^

Papillae are composed of engorged blood vessels with an edematous/inflamed overlying epithelium that varies in size. Adults with inflammation commonly had papillae seen with IVCM. These have been noted in the palpebral conjunctiva of patients with atopic keratoconjunctivitis.^10^ These papillae were not commonly seen among children with active disease. Children did, however, often have structures corresponding to tiny, or micro, papillae: (1) a honeycomb appearance of the tissue with small blood vessels within the cells of the honeycomb and (2) small, superficial vessels perpendicular to the conjunctival surface with prominent surrounding connective tissue. The latter were seen in children with and without clinically apparent inflammation. We suggest that these vessels formed during papillary inflammation and slowly recede once the inflammation has resolved. We would not, therefore, expect this appearance to be found in a population of children from a non-trachoma endemic area.

Another morphologic change seen in active trachoma is the appearance of what has been described as “inflammatory lacunae.”^26^ A feature of active trachoma is thickening of the upper tarsal conjunctiva probably due to edema,^27^ and this probably corresponds to the IVCM finding of these lacunar spaces. This edema has not been observed in histologic studies. However, the processing of histopathology specimens may cause disruption with loss of the spaces.

Changes in cell populations were seen in both children and adults with clinical inflammation. The brightly reflective nuclei seen within and below the epithelium probably belong to inflammatory cells, such as neutrophils or lymphocytes.^7,10,11,28^ This inflammatory infiltrate has been found to be elevated in patients with atopic keratoconjunctivitis compared with controls.^26^ In addition, subconjunctival injection of lipopolysaccharide in rabbit eyes resulted in a significant increase in the infiltrate.^11^ Histopathologic analysis shows a marked inflammatory infiltrate in trachoma of mixed cell types,^23,25,29^ and IVCM shows that this inflammatory infiltrate is generally limited to the superficial 20 μm.

Dendritiform cells have been found with ocular surface IVCM, and studies have been done on their density and distribution in the cornea.^5,30^ We found DCs to be increased in children and adults with inflammation and that they had longer dendritic processes that frequently interdigitated. In addition to being the only antigen-presenting cells that are able to induce primary immune responses, dendritic cells are also important in the regulation of the type of T-cell response and the development of immunologic tolerance.^31,32^ The type of immune response (e.g., a type 1 vs type 2 T-helper response) seems to be important in trachoma in determining whether infection is resolved rapidly or a chronic inflammatory reaction develops.^20,33,34^ The study of dendritic cells in trachoma is also important because their role is likely to be important in the design of any chlamydial vaccine.^35^ However, although IVCM studies have generally labeled DCs as being Langerhans or dendritic cells, immunohistochemistry is needed because other cell types may also have a dendritic morphology.

We are aware of 2 previously published IVCM images of conjunctival scarring. The first of these was from patients with atopic keratoconjunctivitis with an appearance similar to that found in trachoma with organized bands of connective tissue.^10^ The second report shows an unusual honeycomb type appearance that we have not observed elsewhere.^8^ The subepithelial tissue of nonfunctioning blebs shows dense connective tissue with few or no clear spaces, similar to that observed in scarred subjects. Histologic studies of trachomatous scarring have shown the conjunctival stroma to be replaced with compact, largely avascular scar tissue.^36,37^ The tissue organization that we observed with IVCM probably shows collagen fibrils that, in keeping with the histology, may be haphazardly arranged or, in more advanced cases, parallel to each other.

In addition to studying subjects with trachoma, we also examined the palpebral conjunctiva of a larger number of healthy subjects than has previously been done. The appearance of the tarsal conjunctiva in healthy children is largely similar to that of adults. Images of epithelial cells of the tarsal conjunctiva in an oblique view have been published, which are similar to those we found.^7^ It has been reported, using IVCM, that the palpebral conjunctival epithelium is organized in 6 to 7 cell layers.^28^ However, this was also based on an oblique view that may have overestimated the number of layers, which was significantly more than we or previous histologic studies found.^38^ Another published image of the palpebral conjunctival epithelium probably shows images from the marginal portion of the palpebral conjunctiva,^39^ shown from one of our study subjects in Figure 9 (available at http://aaojournal.org).

The “microcystic” spaces that we found in normal palpebral conjunctival epithelium have been observed by other investigators.^8,39^ It is only speculative whether these spaces are actually cystic because it is unknown whether they are lined by epithelium. It is also unknown what these spaces and the debris seen within them represent. It has been suggested that they are occluded goblet cells,^39^ although they seem too large for this and to extend too deep into the tissue. They may represent mucous crypts, which are tubular structures consisting of clusters of goblet cells arranged around a central lumen with an overall diameter of 50 μm.^38^ Kessing^40^ states that there may be stagnation of mucin in these crypts leading to the formation of cysts. Goblet cells are not visible perhaps because they are of a similar reflectivity to the surrounding tissue in the palpebral conjunctiva and the HRT3/RCM is unable to differentiate them.

In vivo confocal microscopy offers a unique opportunity of studying tissue morphology and cellular activity in normal and diseased states. It is not invasive or harmful and can be used repeatedly. A high level of resolution is gained, almost comparable to histologic analysis. Once proficiency with the HRT3/RCM is achieved, images can be obtained within a few minutes, and we have found the machine to be robust and portable. There are, however, some limitations with the technique. The RCM requires contact with the tissue surface, which is not always well tolerated despite topical anesthesia. It can be difficult to satisfactorily position the everted upper lid, especially if the subject has limited mobility. The PMMA cap is currently relatively large and can impede good positioning on the conjunctival surface. The combination of the large cap and very small image area precludes identification of the exact location being imaged. The exact same area cannot, therefore, be imaged sequentially to observe temporal changes. In vivo confocal microscopy in its current state cannot be used in conjunction with tissue/cellular staining, an integral part of histologic-immunohistochemical analysis that yields much valuable information. In vivo confocal microscopy is a relatively new technique, and histologic confirmation of these appearances is needed. We are currently in the process of conducting a case-control study of subjects with trachomatous trichiasis who are undergoing conjunctival biopsies at the time of trichiasis surgery. This will enable a systematic comparison of IVCM and histologic findings.

In conclusion, this article presents descriptive IVCM findings gained from examining a large number of subjects with trachoma compared with healthy control subjects, including children. We also present grading systems for analyzing conjunctival images. We are currently applying this grading system to a large case-control study of trachomatous scarring in which we are correlating confocal microscopy findings with clinical grading. We hope the descriptions presented will be of help in the interpretation of images from patients with a range of conjunctival diseases and further our understanding of trachomatous inflammation, scarring, and blindness.

## Figures and Tables

**Table 1:** Conjunctival Inflammation Grading System for In Vivo Confocal Microscopy

Feature	Grading Description
Inflammatory infiltrate	Mean inflammatory cell density of 3 randomly selected volume scans⁎
DCs	Present or absent: to be present, the mean number of DCs per volume scan needs to be ≥1†
Tissue edema	Present or absent: present if seen in any volume scan
Papillae	Present or absent: present if seen in any volume scan

DC = dendritiform cells.

⁎ Cell density is measured with the RCM software, for which it is suggested that a minimum of 50 cells is counted per field. See text for explanation of why 3 scans are selected. The individual scan with the largest density of cells from within the volume scan is used to calculate the density.

† The largest number of DCs in any individual scan in a particular volume scan is used.
