# Supplementary material for: In Vivo Confocal Microscopy of Trachoma in Relation to Normal Tarsal Conjunctiva
Source: Ophthalmology. 2011 Apr;118(4-2):747–54. doi: 10.1016/j.ophtha.2010.08.029 (PMC3267042; doi:10.1016/j.ophtha.2010.08.029)
Supplement: Fig 2 [file mmc1.pdf]

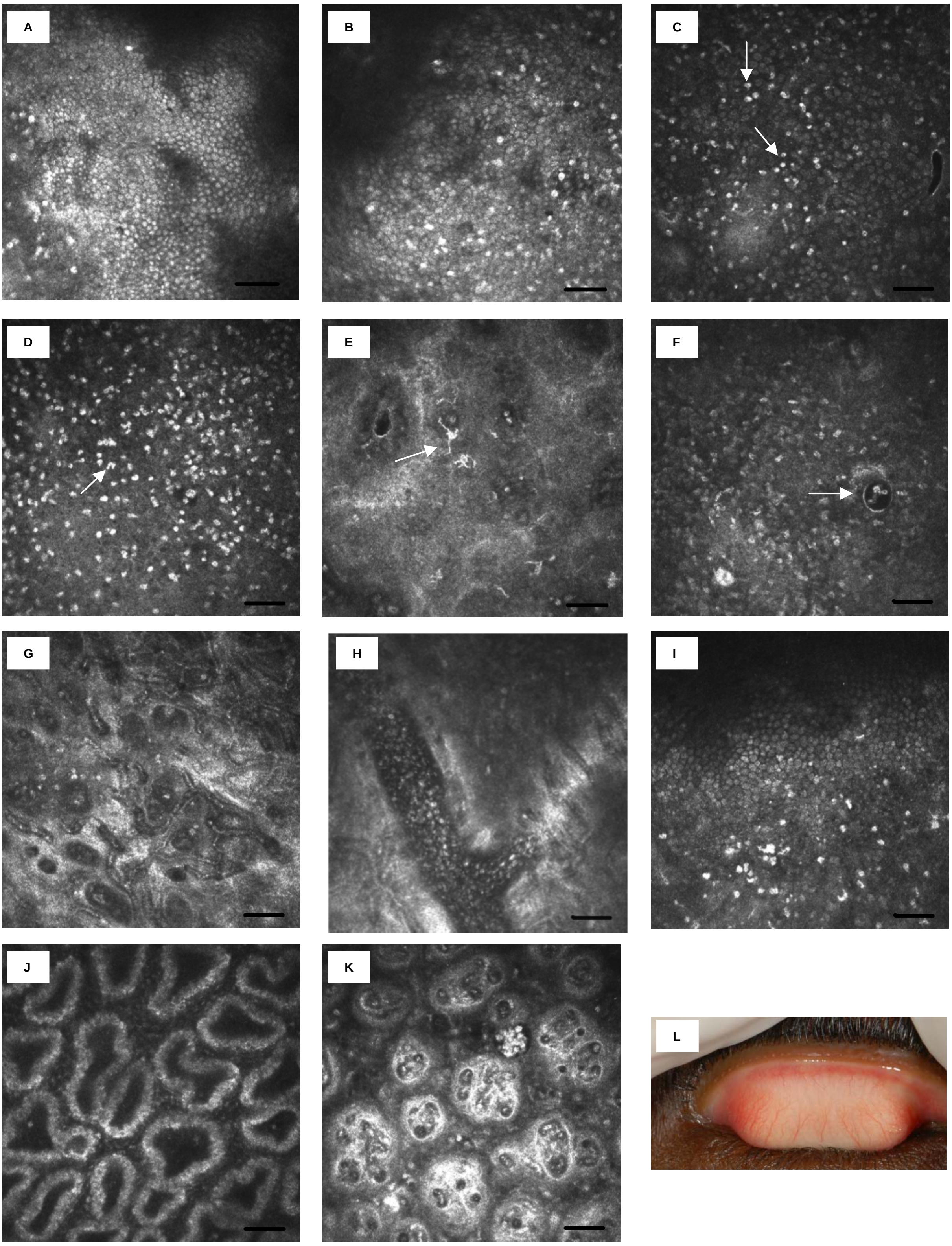

Figure 2. Normal tarsal conjunctiva. Images are 400×400μm with the bar representing 50μm. **A-B,** Superficial epithelial cell nuclei. **C,** Deep epithelial cell nuclei with some brightly reflective inflammatory cell nuclei also visible (arrows). **D,** Inflammatory cell nuclei, note heterogeneity in size and shape (arrow). **E,** Dendritic cells (arrow). **F,** Microcyst (arrow). **G,**

Superficial blood vessels. **H,** Deep blood vessel. **I,** Oblique view. **J,** Meibomian gland acinar units. **K,** Superficial blood vessels perpendicular to the surface with brightly reflective surrounding connective tissue. **L,** Clinical photograph of normal palpebral conjunctiva.
