# Supplementary material for: In Vivo Confocal Microscopy of Trachoma in Relation to Normal Tarsal Conjunctiva
Source: Ophthalmology. 2011 Apr;118(4-2):747–54. doi: 10.1016/j.ophtha.2010.08.029 (PMC3267042; doi:10.1016/j.ophtha.2010.08.029)
Supplement: Fig 3 [file mmc2.pdf]

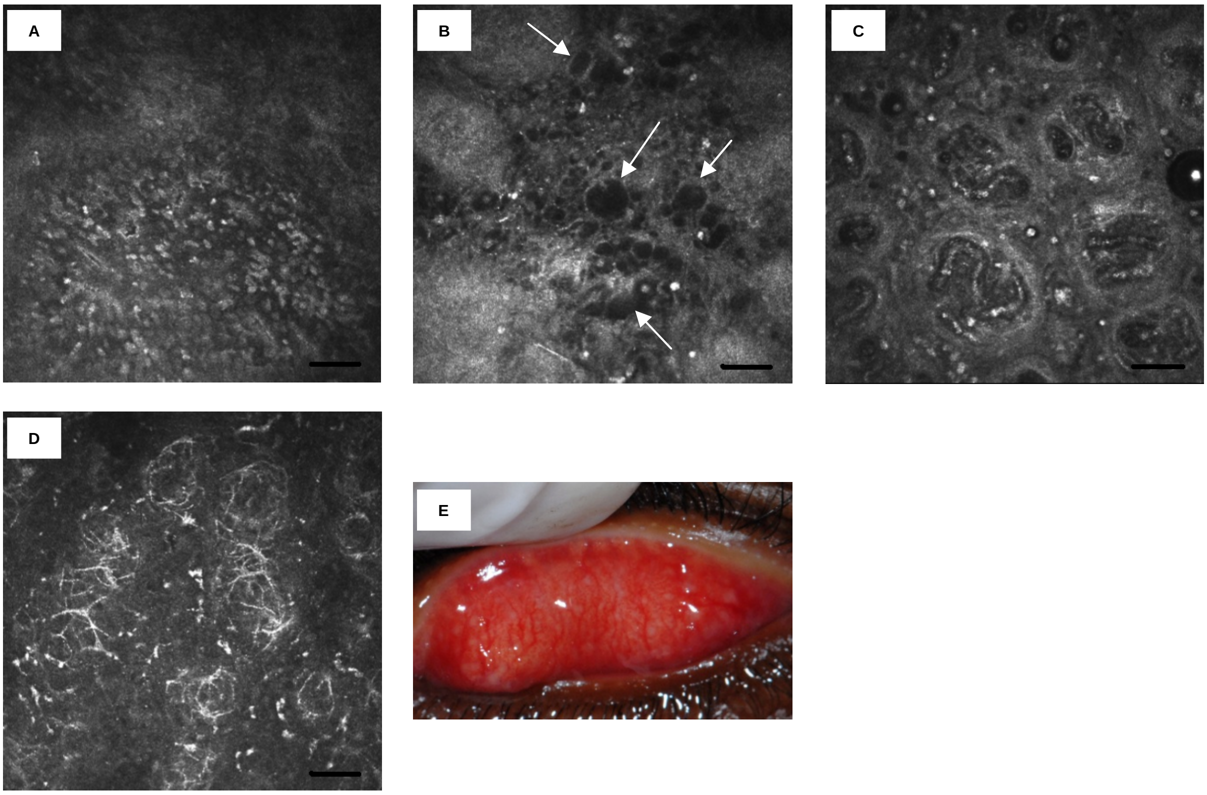

Figure 3. Active disease in children. Images are 400×400µm with the bar representing 50µm. **A,** Follicle at a depth of 20µm. **B,** Cystic lacunae (arrows). The tissue at the edges of this image has some resemblance to early lymphocyte cell aggregates, but no cellularity is seen on further sections at different depths. **C,** Honeycomb with central blood vessels. **D,** Numerous, inter-digitating dendritiform cells. **E,** Clinical photograph of the subject shown in A, note numerous follicles.
