# Supplementary material for: In Vivo Confocal Microscopy of Trachoma in Relation to Normal Tarsal Conjunctiva
Source: Ophthalmology. 2011 Apr;118(4-2):747–54. doi: 10.1016/j.ophtha.2010.08.029 (PMC3267042; doi:10.1016/j.ophtha.2010.08.029)
Supplement: Fig 5 [file mmc3.pdf]

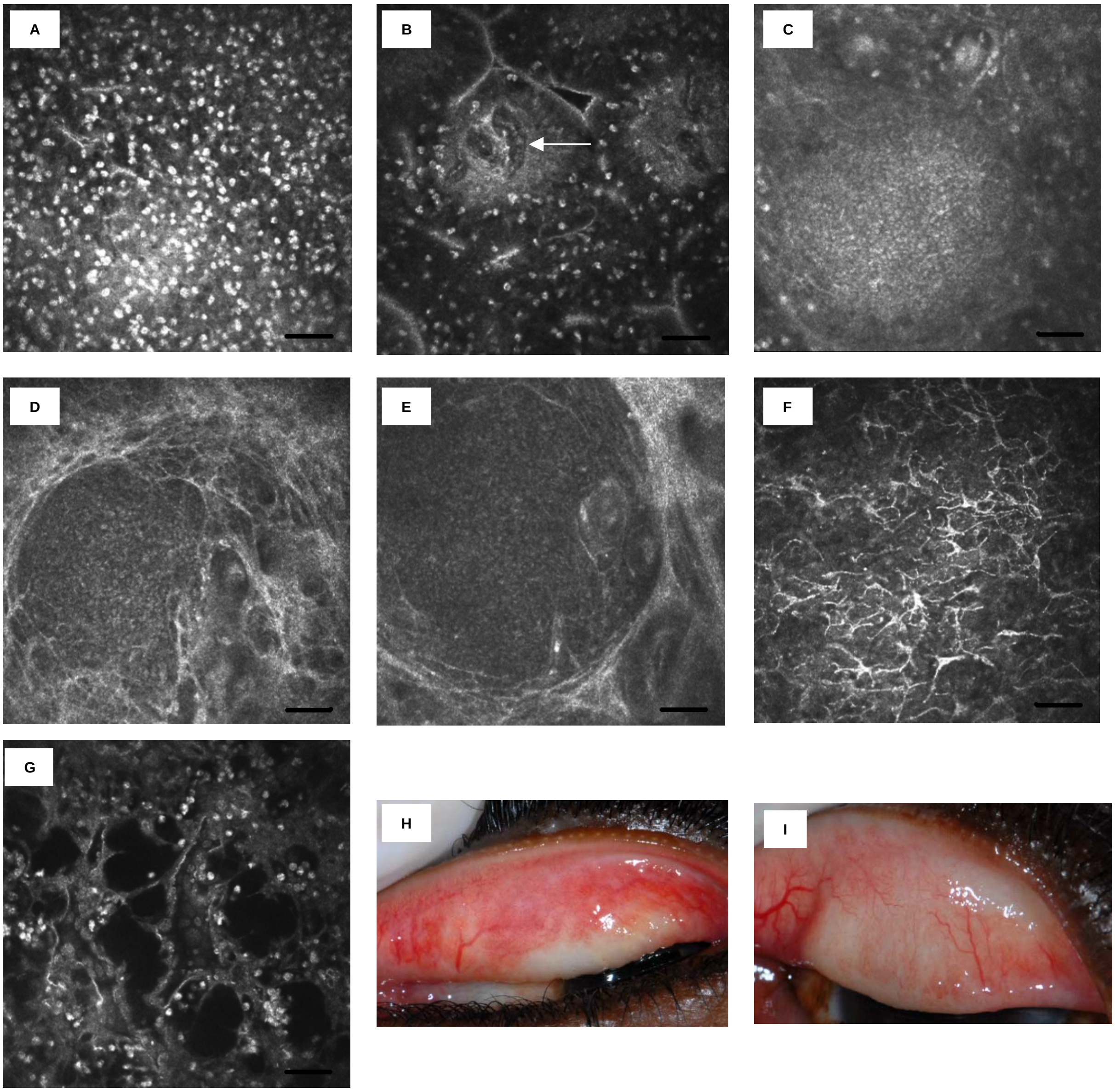

Figure 5. Active disease in adults with conjunctival scarring. Images are 400×400µm with the bar representing 50µm. **A,** Increased inflammatory cell infiltrate. **B,** Papillae with central vessels (arrow). **C-E,** Follicles at various depths. **F,** activated dendritiform cells. **G,** Cystic lacunae. **H,** Clinical photograph of the subject shown in A, note bands of scarring and papillary inflammation with obscuration of deep vessels. **I,** clinical photograph of the subject shown in C, note bands of scarring but lack of prominent follicles.
