# Supplementary material for: In Vivo Confocal Microscopy of Trachoma in Relation to Normal Tarsal Conjunctiva
Source: Ophthalmology. 2011 Apr;118(4-2):747–54. doi: 10.1016/j.ophtha.2010.08.029 (PMC3267042; doi:10.1016/j.ophtha.2010.08.029)
Supplement: Figure 7 [file mmc4.pdf]

Normal: Homogeneous, amorphous appearance, with occasional fine, wispy strands.

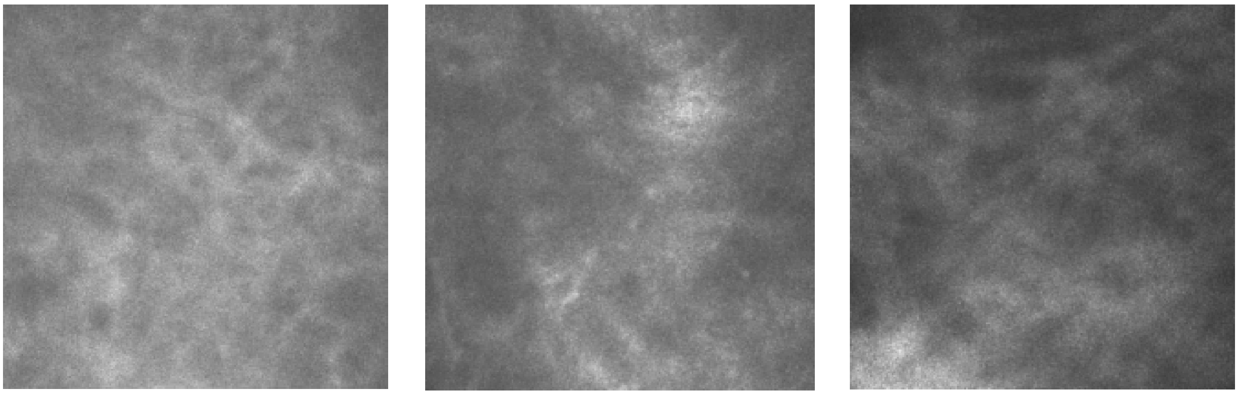

Grade 1: Heterogeneous appearance with poorly defined clumps or bands present.

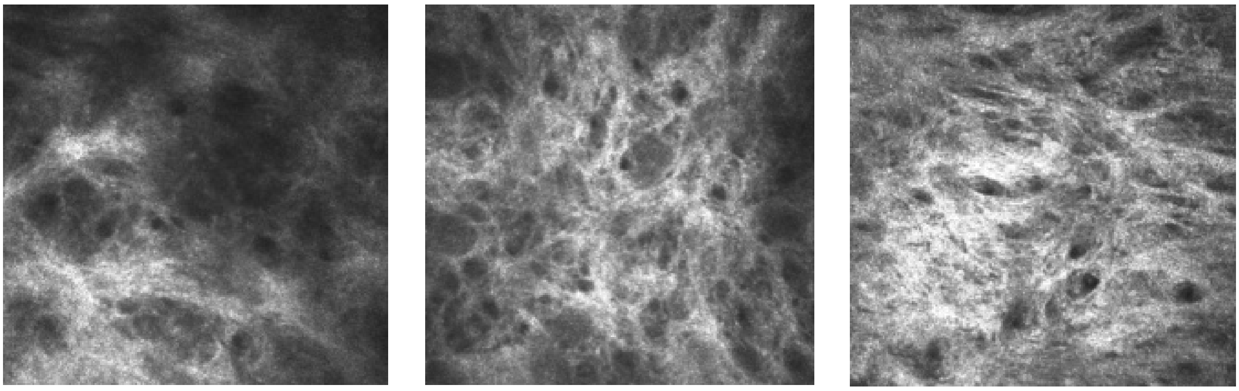

Grade 2: Clearly defined bands of tissue which constitute less than 50% of the area of the scan.

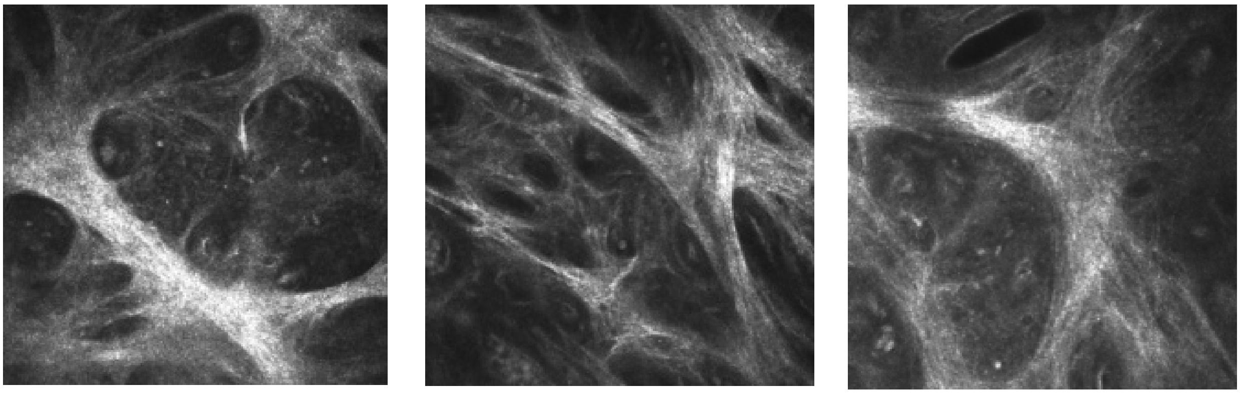

Grade 3: Clearly defined bands or sheets of tissue which constitute 50% or more of the area of the scan and in which striations are visible.

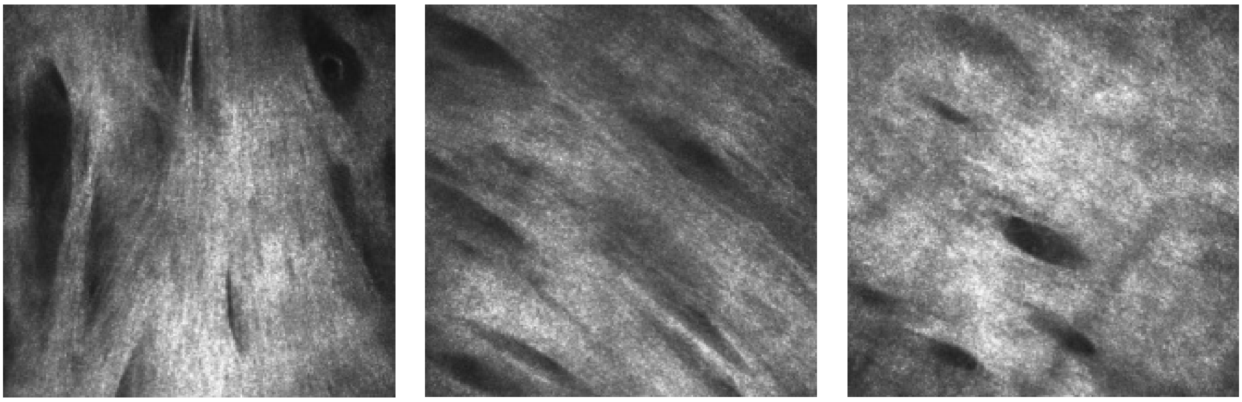

Figure 7. Conjunctival connective tissue organization/scarring grading system for *in vivo* confocal microscopy. Images are 400×400µm. If different grades of scarring are seen within a particular volume scan then the highest grade is recorded. The connective tissue which is graded needs to be separate from that associated with the vascular tissue, if this is not possible then the scan is considered ungradable.
