# Supplementary material for: In Vivo Confocal Microscopy of Trachoma in Relation to Normal Tarsal Conjunctiva
Source: Ophthalmology. 2011 Apr;118(4-2):747–54. doi: 10.1016/j.ophtha.2010.08.029 (PMC3267042; doi:10.1016/j.ophtha.2010.08.029)
Supplement: Fig 8 [file mmc5.pdf]

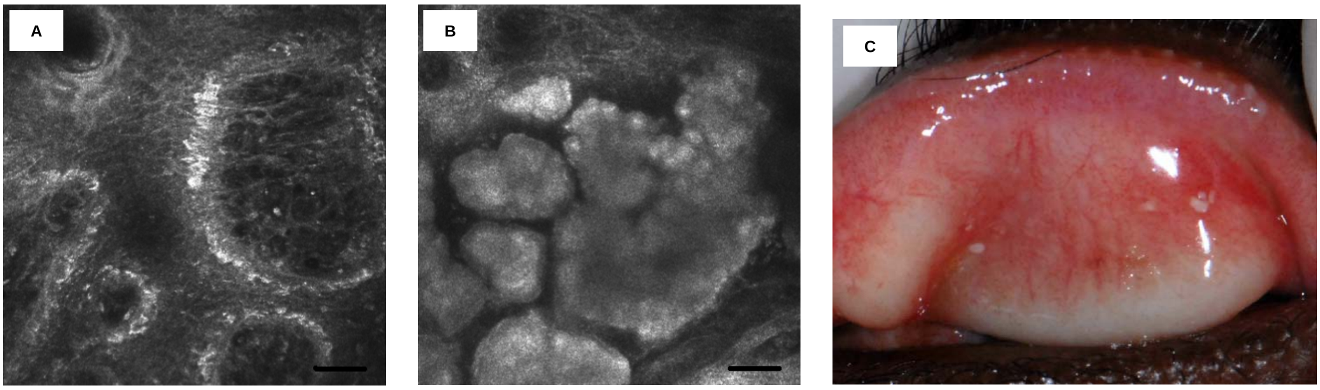

Figure 8. Meibomian gland scarring and concretions in trachoma. Images are 400×400µm with the bar representing 50µm. **A,** Scar tissue in the lumen of meibomian gland acinar units. **B,** concretions. **C,** clinical photograph of the subject shown in B, note concretions.
