# Supplementary material for: In Vivo Confocal Microscopy of Trachoma in Relation to Normal Tarsal Conjunctiva
Source: Ophthalmology. 2011 Apr;118(4-2):747–54. doi: 10.1016/j.ophtha.2010.08.029 (PMC3267042; doi:10.1016/j.ophtha.2010.08.029)
Supplement: Figure 9 [file mmc6.pdf]

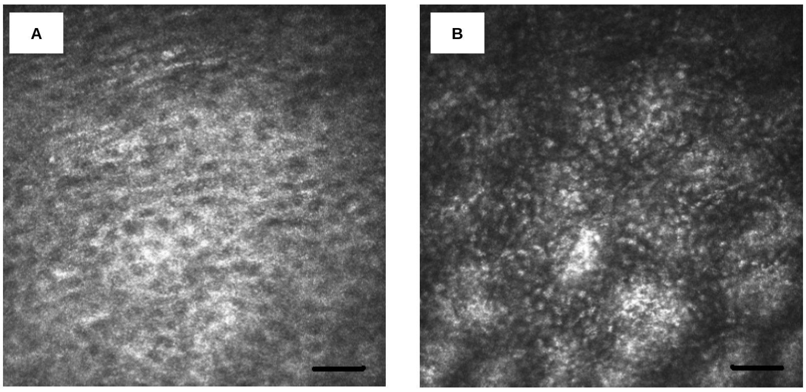

Figure 9. Lid margin epithelium. Images are 400×400µm with the bar representing 50µm. **A,** superficial epithelium. **B,** deeper epithelium.
